# Challenges and Future Prospects on 3D *in-vitro* Modeling of the Neuromuscular Circuit

**DOI:** 10.3389/fbioe.2018.00194

**Published:** 2018-12-11

**Authors:** Maider Badiola-Mateos, Arnau Hervera, José Antonio del Río, Josep Samitier

**Affiliations:** ^1^Institute for Bioengineering of Catalonia-Barcelona Institute of Science and Technology, Barcelona, Spain; ^2^Department of Electronics and Biomedical Engineering, Faculty of Physics, Universitat de Barcelona, Barcelona, Spain; ^3^Department of Cell Biology, Physiology and Immunology, Faculty of Biology, Universitat de Barcelona, Barcelona, Spain; ^4^Centro de Investigación Biomédica en Red sobre Enfermedades Neurodegenerativas, Barcelona, Spain; ^5^Institut de Neurociències de la Universitat de Barcelona, Barcelona, Spain; ^6^Centro de Investigación Biomédica en Red en Bioingeniería, Biomateriales y Nanomedicina, Madrid, Spain

**Keywords:** neuromuscular circuit, compartmentalized microfluidic culture systems (cμFCS), hiPSC, 3D-culture, *in-vitro* models

## Abstract

Movement of skeletal-muscle fibers is generated by the coordinated action of several cells taking part within the locomotion circuit (motoneurons, sensory-neurons, Schwann cells, astrocytes, microglia, and muscle-cells). Failures in any part of this circuit could impede or hinder coordinated muscle movement and cause a neuromuscular disease (NMD) or determine its severity. Studying fragments of the circuit cannot provide a comprehensive and complete view of the pathological process. We trace the historic developments of studies focused on *in-vitro* modeling of the spinal-locomotion circuit and how bioengineered innovative technologies show advantages for an accurate mimicking of physiological conditions of spinal-locomotion circuit. New developments on compartmentalized microfluidic culture systems (cμFCS), the use of human induced pluripotent stem cells (hiPSCs) and 3D cell-cultures are analyzed. We finally address limitations of current study models and three main challenges on neuromuscular studies: (i) mimic the whole spinal-locomotion circuit including all cell-types involved and the evaluation of independent and interdependent roles of each one; (ii) mimic the neurodegenerative response of mature neurons *in-vitro* as it occurs *in-vivo*; and (iii) develop, tune, implement, and combine cμFCS, hiPSC, and 3D-culture technologies to ultimately create patient-specific complete, translational, and reliable NMD *in-vitro* model. Overcoming these challenges would significantly facilitate understanding the events taking place in NMDs and accelerate the process of finding new therapies.

## Introduction

From the physiological and anatomical points of view, the mechanosensory-motor circuit is complex, involving several cell-types with specific natural environments. Traditionally, it has been studied coculturing different cell-types on the same platform from animal origin in 2D (Vilmont et al., 2016; Charoensook et al., 2017; Happe et al., 2017) and 3D (Morimoto et al., 2013; Martin et al., 2015; Smith et al., 2016), or from human origin (Guo et al., 2011; Demestre et al., 2015), or mixed species (Yoshida et al., 2015; Prpar Mihevc et al., 2017). These models provide valuable information in understanding some of the mechanisms underlying the system; but to the date they have not been able to replicate the exact human complexity of physiological functional-units formed by the connection of different cell-types, arising from separated microenvironments. Compartmentalized microfluidic culture systems (cμFCS) (Bhatia and Ingber, 2014) represent an alternative to overcome those problems and, combined with 3D-culture techniques and the use of human induced pluripotent stem cells, they could help recreating neuromuscular physiology of humans *in-vitro*.

This review aims to: (i) provide basic insights about the locomotion circuit and neuromuscular diseases required for its *in-vitro* modeling; (ii) review the breakthrough of bioengineered technologies for neuromuscular-systems; (iii) discuss the limitations and challenges of current study models and future prospects.

## Locomotion Circuit and Neuromuscular Diseases (NMDs)

Locomotion circuit, also known as mechanosensory-motor circuit or reflex-arc circuit, is responsible for executing voluntary, and reflex skeletal-muscle movement, alternating flexion, and extension of the muscle (McCrea, 2001; Purves et al., 2004; Kiehn and Dougherty, 2013). The coordinated-action of cells taking part within is what generates movement: (i) motor-neurons (MN) are in charge of carrying information from the central nervous system to the muscle (Kandel et al., 2013); (ii) sensory-neurons (SN) carry information from the periphery of the body (the muscle in this case) to the central nervous system (Kandel et al., 2013); (iii) interneurons innervate motoneurons and are linked to their pattern of sensory input (Côté et al., 2018); (iv) Schwann cells are small cells that form a myelin-sheath around MN and SN axons that insulates them and enhances signal conduction (Kandel et al., 2013); (v) astrocytes maintain synapses, modulate the transmission of the signal, regulate blood flow, and availability of oxygen, nutrients, and survival factors onto neurons (Rindt et al., 2015); (vi) microglia are phagocytic and immunocompetent cells within the central nervous system, able to induce MN cell-death (Sargsyan et al., 2005; Frakes et al., 2014); (vii) skeletal-muscle cells are multinucleated and elongated cells, with sarcomeric striations that form muscle-fibers distributed in fascicle fashion and are the last executors of voluntary and reflex skeletal-muscle movement (Marieb, 2015; Tortora and Derrickson, 2017).

The events that take part within the neuromuscular-circuit to guide the movement in mammals could be resumed as follows. Once the brain takes the decision of initiating a movement, the signal is transmitted from neocortical projecting neurons through the spinal-cord. Then the spinal-locomotion circuit takes part of guiding the voluntary and reflex skeletal-muscle movement (Purves et al., 2004; Kandel et al., 2013; Tortora and Derrickson, 2017): (1) somatic α-motoneurons (MNs) arising from the ventral-horns of spinal-cord, send the input to the synaptic end-bulbs, triggering calcium flows inwards, and the release of the neurotransmitter acetylcholine (ACh) in the neuromuscular junction (NMJ) between the motoneuron and the motor-end plate of extrafusal muscle-fibers; (2) ACh binds specifically to the skeletal-muscle motor-end plates' ACh-receptors (AChR), inducing contraction of sarcolemma, releasing calcium into the sarcoplasm, that binds to troponin on the thin filaments, facilitating myosin-actin binding, and triggering muscle contraction; (3) intrafusal muscle fibers, located interspersed parallel to extrafusal fibers, change in length as the whole muscle changes; (4) sensory-neurons (SN) sense muscle fiber elongation through muscle-spindle—formed by SN nerve endings wrapped around central areas of intrafusal fibers—, and contraction through Golgi tendon organ—formed by encapsulated structures of collagen fibers located at the joint between muscle fibers and tendons that compress innervating a single SN axon—propagating an impulse signal back to the spinal-cord where is modulated by local interneurons and; (5) γ-motoneurons modulate excitatory input adjusting the contractibility of the muscle-spindle by stimulating intrafusal fibers adapting them to an appropriate length; (6) the integration of both afferent signals from the muscle-spindle and the Golgi tendon organ travels through the spinal-cord to the brain to have awareness of the position of the muscle and movement (muscle extension-flexion state), coordinating movements.

**Figure 1:**
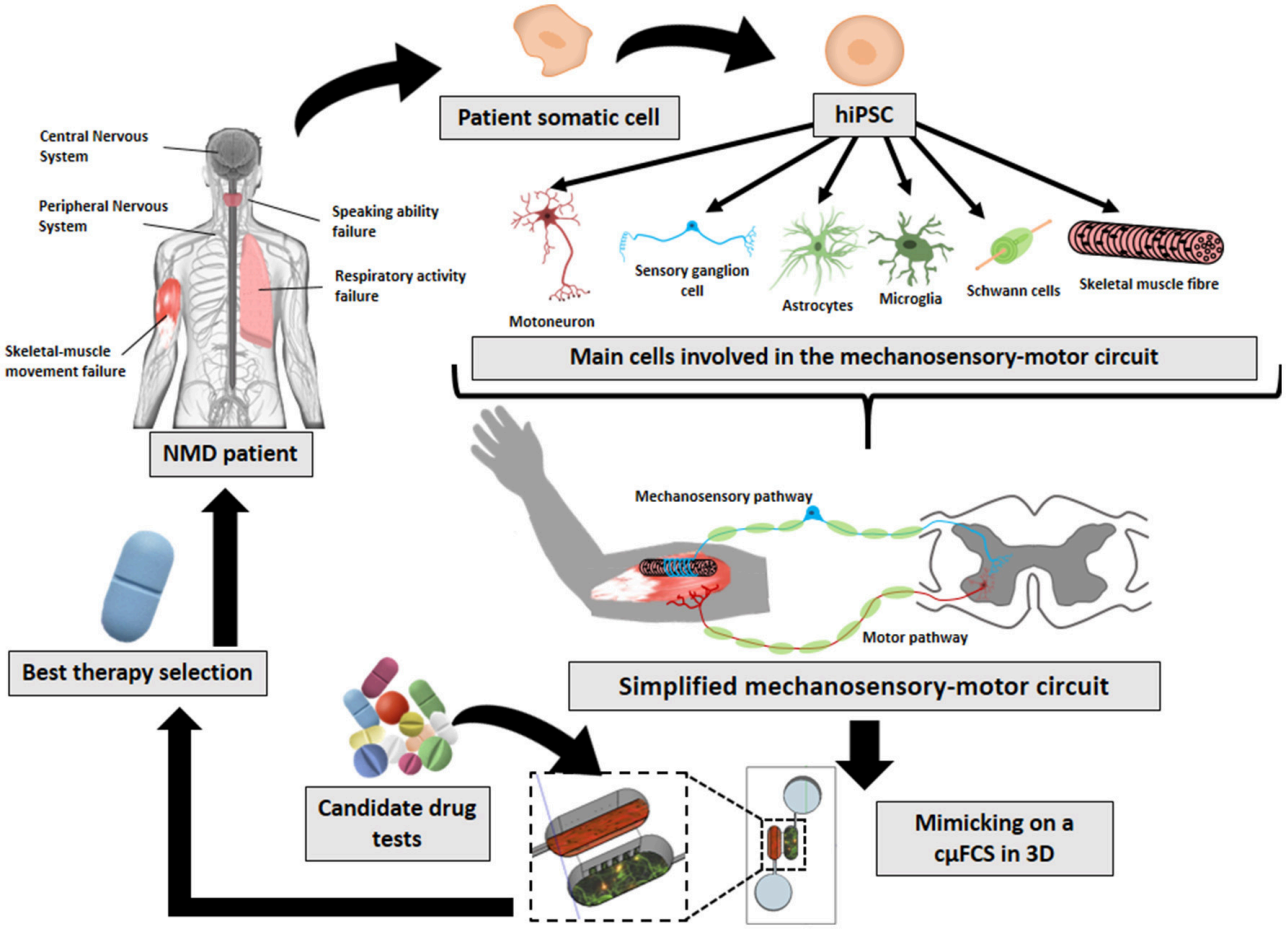
Future prospects on *in-vitro* neuromuscular disease modeling. The implementation and combination of later innovative technologies (hiPSCs, cμFCS, 3D cell-culture) would facilitate the recreation of patient's physiological conditions with his own disease-carrying cells mimicking the mechanosensory-motor circuit in 3D inside a microfluidic compartmentalized device. And finally, it would serve to find specific treatments for each NMD.

Failures in any part of this circuit can hamper coordinated muscle movement and be the cause of neuromuscular diseases (NMDs) or be the consequence that defines their severity (Gogliotti et al., 2012). The term of NMD comprises several diseases with different origins and affectations (such as muscular dystrophy, amyotrophic lateral sclerosis, myasthenia gravis, or spinal muscular atrophy). The effects of NMDs are reflected in the mechanosensory-motor circuit at different cellular levels—including sensory and motor neurons (Jablonka et al., 2006; Gogliotti et al., 2012), Schwann cells (Hunter et al., 2016; Vilmont et al., 2016; Santosa et al., 2018), astrocytes (Rindt et al., 2015), microglia (Frakes et al., 2014; Cooper-Knock et al., 2017), muscle (Martínez-Hernández et al., 2014; Maimon et al., 2018)—, as well as in the connections among them—NMJ (Uzel et al., 2016b; Maimon et al., 2018; Santhanam et al., 2018), muscle spindle (Rumsey et al., 2010; Guo et al., 2017)—, or intraspinal circuits. However, they all share symptoms such as: peripheral hypotonia, muscle weakness, and orthopedic deformities, among others (Bhatt, 2016; Morrison, 2016; Mary et al., 2018). These symptoms impoverishes patient's life-quality (Mary et al., 2018). There is still no treatment for them. Current study models are far from mimicking physiology and therefore are limited on helping to find cures. The technologies here reviewed (Figure 1) aim to help on that direction.

## Mimicking *in-vitro* Human Spinal-locomotion Circuit with hiPSCs in 3D

NMD and NMJ *in-vitro* models have gone through a long evolution history (Thomson et al., 2012): the use of primary cells, cell lines and stem cells; animal-animal cocultures, human-human cocultures, xeno-cocultures; and disease-specific studies. But as reported, most of the research has been done until now by coculturing healthy and diseased cells; primary cells or cell lines with stem cells; and mostly on rodent models or xeno-cultures (human-animal models). However, rodent models offer limited benefit translated into clinic as they do not carry human genetic background. Therefore, personalized medicine needs patient-specific isogenic disease models. In this regard, human induced pluripotent stem cells (hiPSCs) offer the possibility of obtaining different isogenic cell-types from patient's somatic cells, by overexpressing some transcription factors (Takahashi and Yamanaka, 2006), later reviewed (Amabile and Meissner, 2009). They can serve both for creating study models that mimic the physiopathology of the patient, with the further development of future therapeutic transplantation strategies (Su et al., 2013).

The use of induced pluripotent stem cells for disease-modeling, drug-screening, and regenerative therapies of NMDs has widely evolved in the last years (Selvaraj and Perlingeiro, 2018). Nevertheless, few studies have cultured hiPSC-derived motoneurons with hiPSC-derived skeletal-muscle cells *in-vitro* (Demestre et al., 2015; Puttonen et al., 2015; Maffioletti et al., 2018; Osaki et al., 2018). Puttonen et al. (2015) reported a method for the simultaneous differentiation of motoneurons and myotubes from patient-specific hiPSCs, obtaining neuronal differentiation, multinucleated spontaneously contracting myotubes, and functional NMJs on a 2D monolayer. In contrast, Demestre et al. used hiPSCs from healthy donors differentiated separately to motoneurons and myotubes, and subsequently cocultured in 2D. They observed AChR formation in the muscle and neurites outgrowing from motoneurons within the first weeks; but AChR aggregation, maturation of muscle cells, and NMJ formation was not detected until 3 weeks of monolayer cocultures (Demestre et al., 2015).

The advantages and disadvantages between 2D and 3D hiPSC cultures for neurodegenerative disease studies were recently reviewed (Centeno et al., 2018). Briefly, the main outstanding contributions of 3D cultures on NMDs are that: (**i)** it has been proved that 2D monolayers present in some cases altered gene expression whereas 3D cultures display a genotype more relevant to *in-vivo* (Smith et al., 2012; Centeno et al., 2018); (**ii)** cells cultured in 3D acquire more *in-vivo* like phenotype (lower proliferation rate, areas with different levels of oxygen distribution, higher cell-to-cell, and cell-extracellular matrix interactions, increased viability, proliferation, differentiation, and response to stimuli of other cells) (LaPlaca et al., 2010; Antoni et al., 2015; Centeno et al., 2018); (**iii)** some higher-order processes, such as angiogenesis, occur inherently in 3D (Baker and Chen, 2012; Centeno et al., 2018). But so far, only two studies have been able to mimic the NMJ in 3D using hiPSC-derived motoneurons and skeletal-muscle cells *in-vitro* (Maffioletti et al., 2018; Osaki et al., 2018). Maffioletti et al. (2018) used somatic cells from muscular dystrophy patients to create hiPSC-derived isogenic multilineages (skeletal-muscle cells, vascular endothelial cells, pericytes, and motoneurons), subsequently cocultured embedded in fibrin hydrogels. Osaki et al. (2018) created an ALS microphysiological 3D model culturing ALS-hiPSC-derived neural stem cells with hiPSC-derived skeletal-myoblasts embedded in collagen-Matrigel composites.

However, there are still no studies utilizing hiPSC-derived sensory-neurons and hiPSC-derived muscle-cells to mimic the sensory pathway of the spinal reflex-arc circuit. The latest advances on this respect were published by the group of J.J. Hickman using sensory-neurons derived from human neural progenitor cells and intrafusal fibers generated from human skeletal-muscle stem cells, cocultured on a 2D monolayer (Guo et al., 2017).

## Compartmentalized Microfluidic Culture Systems (cμFCS) for Spinal-locomotion Circuit *in-vitro*

Traditional coculture methods do not consider: (i) the different microenvironment requirements of muscle, nerves, and neurons; (ii) the distal connections as they are physically separated *in-vivo*. Furthermore, finding a medium composition compatible for the long-term coculture of both cell-types could be challenging, as several medium components ideal for MN are incompatible with long-term maintenance of skeletal-muscle cells (Thomson et al., 2012; Tong et al., 2014). Hence, a coculture system between neurons and muscle offers limited benefits on mimicking the pathophysiology of NMDs.

The use of compartmentalized microfluidic culture systems (cμFCS) is increasingly growing for neurobiology studies due to the advantages offered compared to classical coculture systems, reviewed in Box 1.

Box 1 **Top 10 advantages of compartmentalized microfluidic culture systems (cμFCS) compared to traditional coculture systems for mimicking spinal-locomotion circuit *in-vitro*.**

1. **Fluidic control**. Compartmentalization enables to control de fluidic environment and provide each cell-type required nutrients to efficiently mature, facilitating survival, functionality, and long-term coculture (Park et al., 2006; Tong et al., 2014).
2. **Experiment feasibility**. Ease to study, enhance, control, and monitor some processes (cell proliferation, differentiation, directional growth, migration, and media diffusion from one compartment to the other) (Kamm and Bashir, 2014; Esch et al., 2015).
3. **Microenvironment spatiotemporal control and monitoring**. Independent manipulation of each compartment cells or extracellular matrix hydrogels could be a critical point to assess some processes. Compartmentalized platforms make possible to dissect molecular and cellular events occurring in somal vs. axonal compartments (effect of compounds, drug-sensitivitiy test, etc.) (Yang et al., 2009; Hur et al., 2011; Zahavi et al., 2015); to track individual axons through microchannels (Hosmane et al., 2011); and to assess axon-specific molecules through immunostaining, protein lisate isolation, and mRNA isolation (Saal et al., 2014; Zahavi et al., 2015).
4. **Customisability of cμFCS designs**. Control over microchannel geometry and dimensions (and therefore sifting of cells or compounds that can pass from one compartment to the other), number of compartments (and therefore number or different microenvironments, if required), compartment division tool (micochannels, microgrooves, membranes), distance between compartments, possibility to include reservoirs, covered, or opened compartments, scalability, and device size (Park et al., 2009; Yang et al., 2009; Hosmane et al., 2010; Uzel et al., 2016b).
5. **Customisable engineering features**. Possibility to integrate and take control over parameters: shear-stress flows (Joanne Wang et al., 2008; Shin, 2009); mechanical (Hosmane et al., 2011), optical (Renault et al., 2015; Jang et al., 2016), and electrical stimuli (Hallfors et al., 2013); topographical cues or micropatterns (Hoffman-Kim et al., 2010); chemical gradients (Uzel et al., 2016a); and sensory systems (Shen et al., 2004; Jeong et al., 2018).
6. **Cost-effectiveness**. Low volume of cells and reagents are required (Millet et al., 2007).
7. **Control over the polarity of neural development**. Culturing somas on one compartment makes possible to take control over axon and dendrite polarity during the development, and hence, to mimic axon injuries and study post-injury regeneration easily (Peyrin et al., 2011; Tong et al., 2015; Renault et al., 2016).
8. **Possibility to mimic neural distal connections**. Neurons growing on one compartment can extend their axons to interact with the other compartment cells, mimicking distal connections (Yang et al., 2009; Zahavi et al., 2015; Maimon et al., 2018).
9. **Possibility to integrate control over the polarity of myocyte differentiation**. Skeletal-muscle cells usually adopt randomized distribution *in-vitro*, whereas the presence of aligned micropatterns for 2D culture, cantilevers for 3D culture, or some stimuli methods (mechanical, electrical, or optical) integrated on the cμFCS can enhance its appropriate differentiation and functionality (Tourovskaia et al., 2008; Hume et al., 2012; Uzel et al., 2014, 2016b).
10. **Better mimicking of physiological conditions and possibility to connect with other microfluidic platforms**. This facilitates the study interactions between different physiological functional-units (Maschmeyer et al., 2015), as well as to integrate blood-flow effects (Maoz et al., 2018), or in a future, to mimic a full human-on-a-chip (Williamson et al., 2013; Kamm and Bashir, 2014), enhancing the development of therapies or diagnostic tools (Esch et al., 2014, 2015; Kamm and Bashir, 2014).

Most relevantly, they enable independent culture conditions for neurons and muscle cells, each supplied by its own microenvironment requirements in different interconnected but fluidically isolated compartments, whilst axons can still go through microgroves connecting cells of both compartments.

First compartmentalized microfluidic culture system (cμFCS) for neurobiology studies on neurotrophic effects of dorsal rood ganglion cells (Campenot, 1977) used Teflon-made open compartments, silicone-glue, and microchannels shattered in glass. Since then, and after soft-lithography fabrication improvements, cμFCS have widely evolved in the last 10 years for spinal-locomotion circuit studies (Supplementary Table 1). Most compartmentalized cocultures are nowadays performed onto polydimethylsiloxane (PDMS)-based platforms with two cellular compartments separated through microchannels or, as described in the only two publications found using a 3D cell coculture, through a gel region (Uzel et al., 2016b; Osaki et al., 2018). Different cell sources are used for MN (predominantly mouse embryonic primary cells) and muscle-cells (using equally rodent hind limb primary skeletal-muscle cells and C2C12 cell-line).

Besides, there are progressively more commercial cμFCS on the market (Zhang and Radisic, 2017), available for neuromuscular and other neurobiology studies in 2D or 3D, connecting two compartments through microchannels, microposts, or membranes, from the following companies: (i) Xona neuron devices (Xona Microfluidics, California), used in some neuromuscular studies (Southam et al., 2013; Blizzard et al., 2015); (ii) ANANDA Neuro-Device and Coculture-Device (Advanced Nano Design Applications Devices, Canada) employed by Magdesian et al. (2016) combined with AFM measurements to study neuronal growth; (iii) OrganoPlates for 3D culture (Mimetas BV, Netherlands) used to differentiate stem cells into neurons in 3D (Moreno et al., 2015) or for high-throughput evaluation of compounds in glia and neuronal 3D culture (Wevers et al., 2016); (iv) AIM 3D culture chips (AimBiotech, Singapore), employed mostly in cancer-research (Jenkins et al., 2018), although with a great potential to be used in neuromuscular studies, as performed by Uzel et al. (2016b) with a similar custom made device; (v) Neural Diode (MicroBrain Biotech, France) to reconstruct oriented neural network monolayer cultures (Peyrin et al., 2011; Deleglise et al., 2014); (vi) Idealized coculture chips (Synvivo, USA), with different options of radial slits or pillars utilized in many cases to mimic the BBB (Prabhakarpandian et al., 2013), or linear slits for compartmentalization purposes.

However, and despite the advantages offered by both hiPSC and cμFCS technologies, at the moment only two studies have attempted to mimic the spinal-locomotion circuit combining both technologies (Osaki et al., 2018; Santhanam et al., 2018). Santhanam et al. (2018) seed healthy donor's hiPSC-derived MN and human skeletal-muscle fibers in 2D, for dose-evaluation study of toxins affecting the NMJ. Osaki et al. create an ALS microphysiological 3D *in-vitro* study-model and compare it with a healthy model (muscle contraction, recovery, and response to drugs administered via endothelial cell barrier).

## Limitations of Current Study Models and Main Challenges

### Combination of hiPSCs, cμFCS, and 3D-Culture Technologies

Uzel et al. (2016b) combine cμFCS with 3D cell-culture techniques for neuromuscular studies. Additionally, Santhanam et al. (2018) have attempted to mimic the spinal-locomotion circuit combining hiPSCs and cμFCS technologies. And Maffioletti et al. (2018) perform 3D culture of hiPSCs for NMD studies. But thus far, there is one single study combining 3D culture, hiPSCs and cμFCS as NMD models (Osaki et al., 2018). The three innovative technologies offer advantages (Figure 1), but the novelty itself comes with the challenge of developing, tuning, and implementing them together in a unique and biologically reproducible functional platform.

### Consideration of Main Actors and Roles From the Spinal-Locomotion Circuit

The co-culture of main cell-types participating in the spinal-locomotion circuit is mandatory to provide a native microenvironment, including the inherent release of growth-factors, as well as to support the viability and maturation of both muscle and neurons, and axon elongation of MNs (Gingras et al., 2008). For instance, spinal MNs cannot achieve proper maturation even after long-term maintenance, unless cultured with muscle-cells, and Schwann cells, as previously reviewed (Bucchia et al., 2018).

Besides, both SN and MN could be altered in particular NMDs (Jablonka et al., 2006; Rumsey et al., 2010; Guo et al., 2017), but not being many available studies focused on the muscle spindle (Taylor et al., 2005; Dagberg and Alstermark, 2006; Rumsey et al., 2010; Bewick and Banks, 2015; Matthews, 2015; Guo et al., 2017) challenges the task of mimicking and characterizing the mechanosensory spinal-locomotion circuit. Additionally, glial cells are also affected and involved in several neuromuscular pathologies (Lobsiger and Cleveland, 2007; Vilmont et al., 2016; Bucchia et al., 2018). Yet, most publications do not consider them.

Most *in-vitro* NMD studies are focused on the α-MN-muscle connection (Supplementary Table 1), very few on the γ-MN-muscle connection (Colón et al., 2017), and some on the SN-muscle connection (Taylor et al., 2005; Dagberg and Alstermark, 2006; Rumsey et al., 2010; Bewick and Banks, 2015; Matthews, 2015; Guo et al., 2017; Levin et al., 2017) and fewer on the internal SN-MN connection (Schwab and Ebert, 2014). But there is still very little known about what happens beyond those connections, to what extent cell-strategies for synaptic-specificity contribute on the formation of a functional connection (Fukuhara et al., 2013; Maimon et al., 2018).

Studying parts of the circuit cannot provide a comprehensive and complete view of the pathological process, and functional alterations occurring within it. The big challenge that remains out there on NMD studies is the modeling of the whole spinal-locomotion circuit with all cell-types involved, and the evaluation of independent and interdependent roles of each part of the circuit on the development of a particular NMD.

### Modeling Neurodegeneration in NMDs

In a broad sense, neurodegeneration is a process characterized by the progressive functional loss of a population of neurons by intrinsic cell-death or the loss of support cells (i.e., oligodendrocytes or astrocytes). Most NMDs are characterized by this devastating phenomenon (i.e., motoneuron diseases: amyotrophic lateral sclerosis, or spinal muscular atrophy, etc.).

Indeed, studying only functional changes in MNs cannot give a comprehensive and complete picture of the process as it is also regulated by non-neuronal cells (Lobsiger and Cleveland, 2007; Bucchia et al., 2018; Maimon et al., 2018). For instance, a recent study performed by Maimon et al. (2018) demonstrated that axon degeneration only occurred with both MN and muscle cells carrying the genetic mutation indicative of the disorder. Furthermore, the morphology of spinal MN axons *in-vitro* differs from the one presented *in-vivo*: contrary to *in-vivo*, axonal terminals *in-vitro* manifest growth-cones, and are prone to regenerate and lengthen in response to neurotrophic factors required for the *in-vitro* maintenance of the culture (Bucchia et al., 2018). On top of that, another caveat is the fact that extrapolating effects of short-term studies to longer-term disease processes is often not correlated. Therefore, mimicking neurodegeneration response *in-vitro* as it occurs *in-vivo* endures to this day as a challenge. And consequently, there is no effective treatment for neurodegenerative NMDs to promote axonal regeneration yet.

## Conclusions and Future Prospects

Finding causes and treatment for NMD requires an accurate modeling of the microphysiological conditions that the patient is suffering. But reproducing the complete spinal-locomotion (reflex-arc) circuit *in-vitro* is very complex. Later progresses in neuromuscular-mimicking *in-vitro* systems, have been achieved incorporating increasingly evolving technologies of hiPSCs, cμFCS, and 3D cell-culture techniques here reviewed. The combination of novel technologies in the proper manner has proved to result in the acquisition of more reliable results (Uzel et al., 2016b; Maffioletti et al., 2018; Osaki et al., 2018; Santhanam et al., 2018). But there is still room for improvement. Future studies should focus on addressing unsolved questions related to: mimicking the whole spinal-locomotion circuit (including all cell-types involved, as well as evaluating the independent and interdependent roles of each one), defining the specific role of the factors that determine the NMD and their severity; mimicking neurodegeneration processes; and above all, finding treatments for NMD.

## Author Contributions

MB-M wrote the manuscript with contributions from AH, JdR, and JS.

### Conflict of Interest Statement

The authors declare that the research was conducted in the absence of any commercial or financial relationships that could be construed as a potential conflict of interest.
