# Supplementary material for: Challenges and Future Prospects on 3D in-vitro Modeling of the Neuromuscular Circuit
Source: Front Bioeng Biotechnol. 2018 Dec 11;6:194. doi: 10.3389/fbioe.2018.00194 (PMC6297173; doi:10.3389/fbioe.2018.00194)
Supplement: Supplementary file 1 [file Table_1.docx]

# Supplementary material

| **Table 1. Summary of the last 10 years evolution of publications using compartmentalised microfluidic culture systems (cµFCS) for the study of spinal-locomotion circuit (both motor and mechanosensory pathways).** This review is limited to publications employing motoneurons or sensory neurons and skeletal-muscle, from rodent or human origin.  **N/A** = Not applicable. No publications were found utilising cµFCS to study the mechanosensory pathway.  **H/H** = healthy neurons and healthy muscle cells utilised in the study.  **ALS** = amyotrophic lateral sclerosis  **LxWxH** = length x width x height. | | | | | | |
| --- | --- | --- | --- | --- | --- | --- |
|  | **Neuron cell source** | **Muscle cell source** | **2D or 3D coculture** | **Disease?** | **cµFCS device fabrication and compartment separation method** | **Reference** |
| **Mechanosensory pathay** | N/A | N/A | N/A | N/A | N/A | N/A |
| **Motor pathway** | embryonic rat (E15) primary MNs | postnatal rat (P2) hind limb primary skeletal-muscle cells | 2D | H/H | Xona microfluidics commercial device | (Southam et al., 2013) |
|  | mouse ESC-derived embryoid bodies differentiated to MNs | mouse myoblast C2C12 cell line | 2D | H/H | Custom made PDMS device.  Microchannel dimensions (LxWxH): 500 µm x 10 µm x 2.5 µm | (Hyun Sung Park et al., 2013) |
|  | mouse embryonic (E13.5) MNs | mouse myoblast C2C12 cell line | 2D | H/H | Custom made PDMS device.  Microchannel dimensions (LxWxH): 1mm x 10µm x 2.7µm | (Tong et al., 2014) |
|  | mouse embryonic (E13.5) MNs | mouse myoblast C2C12 cell line | 2D | H/H | Xona microfluidics commercial device | (Blizzard et al., 2015) |
|  | mouse embryonic (E11-E12) MNs | adult mice hind limb primary skeletal-muscle cells | 2D | H/H | Custom made PDMS device.  Microchannel dimensions (LxWxH): 400 µm x 15 µm x 5 µm | (Zahavi et al., 2015) |
|  | mouse embryonic (E11.5-E12.5) MNs | adult (>P30) mice hind limb primary skeletal-muscle cells | 2D | H/H | Custom made PDMS device.  Microchannel dimensions (LxWxH): 400 µm x 15 µm x 5 µm | (Ionescu et al., 2016) |
|  | mouse embryonic stem cell line HBG3 differentiated into light activatable MN | mouse myoblast C2C12 cell line | 3D | H/H | Custom made PDMS device.  A gel region separates both compartments 1mm. | (Uzel et al., 2016b) |
|  | hiPSC-derived MN | human skeletal muscle myoblastas cell line | 2D | H/H | Custom made PDMS device.  Microchannel dimensions (LxWxH): 400 µm x 10 µm x 3.5 µm | (Santhanam et al., 2018) |
|  | human spinal cord stem cell-derived MNs |  |  |  |  |  |
|  | mouse embryonic (E12.5) MNs | adult (P60) mice hind limb primary skeletal-muscle cells | 2D | H/H | Custom made PDMS device.  Microchannel dimensions (LxWxH): 400 µm x 15 µm x 5 µm. | (Maimon et al., 2018) |
|  | SOD1G93A or HB9::GFP mouse embryonic (E11.5) ventral horn explants | adult (P60) SOD1G93A mice hind limb primary skeletal-muscle cells |  | ALS |  |  |
|  | MN spheroids formed from human embryonic stem cell–derived neural stem cells | hiPSC-derived skeletal myoblasts | 3D | H/H | Custom made PDMS device.  A gel region separates both compartments 700 µm. | (Osaki et al., 2018) |
|  | MN spheroids formed from ALS-patient hiPSC-derived neural stem cells |  |  | ALS |  |  |
